# Deep Reinforcement Learning for Secure and Low-Latency Communications in UAV-Mounted STAR-RIS Assisted Urban Vehicular Networks

**DOI:** 10.3390/s26113469

**Published:** 2026-05-31

**Authors:** Jian Tang, Jun Yuan, Hu Zhao, Mengxiang Chen, Yi Peng

**Affiliations:** 1 School of Artificial Intelligence, Shaoyang Industry Polytechnic College, Shaoyang 422000, China; jian_tang2021@126.com (J.T.); mengxiang_chen@126.com (M.C.); 2 Faculty of Information Engineering and Automation, Kunming University of Science and Technology, Kunming 650000, China; 12309214@kust.edu.cn

**Keywords:** vehicular networks, UAV, STAR-RIS, secure communications, low-latency transmission, deep reinforcement learning

## Abstract

This paper investigates secure and low-latency communications in UAV-mounted simultaneously transmitting and reflecting reconfigurable intelligent surface (STAR-RIS)-assisted urban vehicular networks, where severe blockage, high vehicle mobility, eavesdropping threats, and delay-sensitive traffic services coexist. In the considered system, the UAV is used not only as an aerial carrier for the STAR-RIS but also as a mobile intelligent control node that can dynamically adjust its horizontal aerial position according to vehicle distribution, blockage conditions, and eavesdropping threats. First, a UAV-STAR-RIS-assisted vehicular communication system model is developed by jointly considering urban blockage, vehicle mobility, passive eavesdropping attacks, queueing dynamics, and UAV flight constraints. Then, a high-dimensional, non-convex, and strongly coupled dynamic optimization problem is formulated to maximize the long-term average secure and low-latency utility through the joint optimization of the UAV trajectory, the STAR-RIS transmission–reflection partition ratio, the phase-shift matrices, and the transmit power allocation. Furthermore, the problem is modeled as a Markov decision process with continuous state and action spaces, and a hierarchical constrained soft actor–critic (HC-SAC)-based joint control algorithm is proposed to enable adaptive UAV movement, STAR-RIS configuration, and power control in complex dynamic environments. Simulation results demonstrate that the proposed method outperforms DDPG and several structural benchmark schemes. In the representative evaluation, the proposed HC-SAC achieves an average delay of 10.85 slots and a secrecy outage probability of 0.7160, compared with 11.72 slots and 0.8501 for PPO, and 11.94 slots and 0.8599 for DDPG. Although PPO provides the highest average secrecy rate and successful service ratio, the proposed method still maintains a competitive secure communication capability and service reliability. A normalized composite utility analysis further shows that HC-SAC attains the highest utility value of 0.9254, indicating a more favorable security–latency trade-off in complex urban vehicular scenarios.

## 1. Introduction

Integrated sensing and communications (ISAC) has emerged as a promising technology for next-generation wireless systems, since it enables spectrum sharing and functional integration between communication and sensing tasks. Meng et al. [1] discussed the opportunities and challenges of cooperative ISAC networks, and pointed out that network-level cooperation can expand sensing and communication coverage and provide additional degrees of freedom for ISAC design. For vehicular scenarios, Liu et al. [2] investigated energy-efficient computation offloading and resource allocation in ISAC-aided 6G V2X networks, showing that ISAC can support both communication services and computation-intensive vehicular applications. Cheng et al. [3] further studied ISAC for vehicular communication networks and highlighted its potential in supporting environmental perception, target awareness, and reliable vehicular information exchange. These studies indicate that ISAC is particularly attractive for urban vehicular networks, where high-rate communication, environmental sensing, and service awareness are required simultaneously.

However, urban vehicular environments also bring severe challenges to ISAC-enabled V2X transmission. Roadside buildings, overpasses, and dense traffic flows may cause frequent blockage and rapid channel fluctuations, which seriously degrade communication reliability and coverage continuity. Meanwhile, vehicular services such as cooperative driving, hazard warning, and real-time control are delay-sensitive, making low-latency transmission an essential requirement. More importantly, the broadcast nature of wireless channels makes vehicular messages vulnerable to interception. Yu et al. [4] studied movable-antenna-aided secure V2X communications from an ISAC perspective, indicating that mobility-aware antenna reconfiguration can improve secure transmission performance. Hasan et al. [5] provided a comprehensive study on securing V2X communication platforms and summarized key security threats in vehicular networks. Gyawali et al. [6] reviewed the major challenges and solutions for cellular-based V2X communications, including reliability, latency, and resource management issues. Hakeem and Kim [7] further surveyed machine learning, federated learning, and edge AI techniques for V2X intrusion detection. Although these works have advanced secure and reliable V2X communications, the joint design of physical-layer security, low-latency service, and UAV-assisted intelligent propagation control remains insufficiently explored.

To improve propagation quality in blocked environments, reconfigurable intelligent surfaces (RISs) have been widely recognized as an effective means of reshaping wireless channels. ElMossallamy et al. [8] summarized the principles, challenges, and opportunities of RIS-assisted wireless communications, showing that RIS can enhance link quality by tuning the electromagnetic responses of passive elements. However, conventional RIS mainly relies on half-space reflection, which limits its service capability when vehicles are distributed on both sides of urban roads. To address this limitation, Mu et al. [9] investigated simultaneously transmitting and reflecting RIS (STAR-RIS)-aided wireless communications, where the incident signal can be simultaneously transmitted and reflected to provide full-space coverage. In the V2X context, Aung et al. [10] proposed a deep reinforcement learning-based joint spectrum allocation and configuration design method for STAR-RIS-assisted V2X communications, demonstrating the potential of STAR-RIS in dynamic vehicular environments. Nevertheless, most existing STAR-RIS-assisted V2X studies mainly focus on spectrum allocation or configuration design, while secure low-latency communication with UAV-mounted STAR-RIS remains less investigated.

Although STAR-RIS offers stronger propagation control capability, fixed roadside or building-mounted deployment still suffers from limited coverage adaptability and poor flexibility in highly dynamic vehicular scenarios. In contrast, unmanned aerial vehicles (UAVs) can provide agile aerial deployment and favorable line-of-sight links. Andreou et al. [11] studied UAV-assisted roadside units for V2X connectivity using Voronoi diagrams in 6G+ infrastructures, showing that UAVs can improve vehicular connectivity through flexible aerial assistance. Peng et al. [12] investigated a UAV-borne STAR-RIS-assisted non-orthogonal multiple access system and developed a joint power allocation algorithm, verifying the feasibility of mounting STAR-RIS on UAV platforms. These studies show that the combination of UAV mobility and STAR-RIS programmability can provide adaptive communication support for vehicular networks. However, existing UAV-assisted or UAV-borne STAR-RIS studies have not fully considered the coupling among UAV trajectory, STAR-RIS transmission–reflection partitioning, phase-shift control, eavesdropping threats, and queue-aware latency.

Several recent studies have further investigated UAV-RIS communications and learning-based UAV network optimization. Nakazato et al. [13] proposed a multi-agent reinforcement learning method for resilient UAV ad hoc backhaul networks, where multiple UAVs collaboratively adjust their deployment to improve coverage and connectivity. This work demonstrates the applicability of reinforcement learning to dynamic low-altitude UAV networks, but it does not consider RIS/STAR-RIS-assisted physical-layer transmission or secure vehicular services. Li et al. [14] studied an RIS-assisted UAV communication system and jointly optimized the UAV trajectory and RIS passive beamforming to maximize the average achievable rate. Yang et al. [15] analyzed the performance of RIS-assisted dual-hop UAV communication systems and derived analytical expressions for outage probability, average bit-error rate, and average capacity, confirming the coverage and reliability gains introduced by RIS. Liu et al. [16] further proposed a machine-learning-empowered UAV-RIS framework, where UAV movement, RIS phase shifts, power allocation, and decoding order were jointly designed. In terms of secure UAV communication, Li et al. [17] investigated robust secure UAV communications with the aid of RIS and jointly optimized the UAV trajectory, RIS passive beamforming, and transmit power under imperfect eavesdropping CSI. Shi et al. [18] provided a comprehensive survey of RIS-aided cell-free massive MIMO systems for 6G and summarized different RIS architectures and applications, including STAR-RIS and UAV integration. Although these studies provide important foundations for UAV-RIS communication, RIS-assisted security, and learning-based UAV control, they mainly focus on conventional reflecting RISs, fixed RIS deployment, or rate/security-oriented optimization. The joint design of UAV-mounted STAR-RIS, high-mobility vehicular users, queue-aware low-latency services, and passive eavesdropping defense remains insufficiently studied.

Meanwhile, physical-layer security and intelligent resource control in RIS-assisted V2X networks have drawn growing attention. Saikia et al. [19] proposed a PPO-based method for RIS-assisted full-duplex 6G-V2X communications, showing that reinforcement learning can support dynamic RIS configuration and resource allocation. Long et al. [20] studied deep reinforcement learning for ISAC in RIS-assisted 6G V2X systems, where sensing and communication performance are jointly optimized. Wang et al. [21] investigated physical-layer security enhancement using artificial noise in C-V2X networks, demonstrating that artificial noise can help suppress eavesdropping. De Lima et al. [22] considered broadband beamforming and jamming mitigation in V2X scenarios, which is related to anti-interference and robust V2X transmission. Shang et al. [23] further studied energy-efficient and intelligent ISAC in V2X networks using spiking-neural-network-driven DRL. These studies provide useful references for secure, intelligent, and ISAC-oriented V2X communications. Nevertheless, they mainly focus on fixed RIS, conventional V2X infrastructure, artificial-noise-aided security, or ISAC resource allocation, while the joint security–latency optimization of UAV-mounted STAR-RIS-assisted vehicular networks remains an open problem.

Deep reinforcement learning (DRL) provides a natural tool for the considered dynamic optimization problem. Since the system involves time-varying vehicle positions, queue evolution, eavesdropper distribution, UAV motion, and STAR-RIS configuration, the resulting optimization problem is high-dimensional, strongly coupled, and non-convex. Conventional model-based optimization methods often incur high computational complexity and are not well-suited for real-time adaptation in fast-changing vehicular scenarios. Li et al. [24] proposed a physical-layer eavesdropping defense scheme for V2X based on an improved SAC algorithm, indicating that SAC-type methods are effective for secure V2X decision-making. Amudha et al. [25] studied a hyperparameter-tuned PPO-based federated DRL method for efficient V2X resource allocation, showing the applicability of PPO in V2X resource management. Mlika and Cherkaoui [26] applied DDPG to minimize the age of information in cellular V2X communications, demonstrating the suitability of deterministic policy gradient methods for continuous vehicular control problems. However, directly applying conventional SAC, PPO, or DDPG to the considered scenario is still challenging because UAV movement, STAR-RIS partitioning, phase-shift configuration, transmit power allocation, secrecy constraints, and latency requirements are tightly coupled.

Motivated by the above observations, this paper investigates secure and low-latency communications in UAV-mounted STAR-RIS-assisted urban vehicular networks. Different from existing studies, the UAV in this work is used not only as an aerial carrier for the STAR-RIS but also as a mobile intelligent control node that can dynamically adjust its trajectory according to vehicle distribution, blockage conditions, queue states, and eavesdropping threats. Meanwhile, the STAR-RIS transmission–reflection partition ratio, phase-shift matrices, and transmit power allocation are jointly optimized with the UAV trajectory. To better handle the coupled control variables and the stringent security–latency trade-off, we formulate a long-term utility maximization problem and develop a hierarchical constrained soft actor–critic (HC-SAC)-based joint optimization method. Specifically, the original problem is first transformed into a Markov decision process with continuous state and action spaces, and then the UAV trajectory, STAR-RIS transmission–reflection partition ratio, phase-shift matrices, and transmit power allocation are jointly optimized through hierarchical and constraint-aware policy learning.

To further clarify the technical differences between this work and existing studies, Table 1 summarizes representative related works in terms of the considered scenario, UAV mobility, RIS/STAR-RIS configuration, physical-layer security, queue-aware latency, learning-based optimization, hierarchical control, and constraint-aware learning.

As shown in Table 1, existing UAV-RIS studies mainly focus on trajectory design, passive beamforming, performance analysis, or secrecy-rate maximization with conventional reflecting RISs. Existing STAR-RIS-assisted V2X works mainly consider fixed surface deployment and spectrum/resource allocation, while the UAV-mounted STAR-RIS architecture has not been sufficiently investigated for secure and low-latency vehicular communication. Moreover, existing DRL- or SAC-based V2X studies rarely integrate UAV trajectory control, STAR-RIS transmission–reflection partitioning, phase-shift design, power allocation, physical-layer security, and queue-aware latency into a unified constrained learning framework. Therefore, the proposed HC-SAC method differs from existing studies by jointly exploiting UAV mobility, STAR-RIS full-space reconfiguration, and hierarchical constraint-aware policy learning.

Compared with the existing literature, the main differences of this work are threefold. First, unlike conventional RIS-assisted or STAR-RIS-assisted V2X studies that mainly consider fixed surface deployment, this paper investigates a UAV-mounted STAR-RIS architecture, where UAV mobility provides additional spatial degrees of freedom for adaptive urban vehicular coverage. Second, unlike existing secure V2X or UAV-RIS works that mainly focus on artificial noise, beamforming, secrecy-rate maximization, or resource allocation, this paper jointly considers passive eavesdropping threats, queue-aware low-latency service, and UAV flight constraints. Third, unlike existing DRL-based V2X optimization methods, the proposed HC-SAC framework jointly learns UAV trajectory control, STAR-RIS transmission–reflection partitioning, phase-shift configuration, and transmit power allocation under coupled security and latency constraints.

The main contributions of this paper are summarized as follows:

- A UAV-mounted STAR-RIS-assisted urban vehicular communication framework is established by jointly considering urban blockage, dynamic vehicle mobility, passive eavesdropping threats, queueing delay, and UAV mobility constraints.
- A long-term, secure, and low-latency utility maximization problem is formulated to jointly optimize the UAV trajectory, the STAR-RIS transmission–reflection partition ratio, the phase-shift matrices, and the transmit power allocation, resulting in a high-dimensional and strongly coupled continuous-control problem.
- A hierarchical constrained soft actor–critic-based joint optimization algorithm is proposed to address the above problem. The developed method improves the adaptability of UAV-mounted STAR-RIS control in dynamic vehicular scenarios and enhances the trade-off between secrecy performance and delay efficiency.
- Simulation results demonstrate that the proposed method outperforms DDPG and all structural benchmark schemes. Compared with PPO, it achieves lower delay and lower secrecy outage probability while maintaining a competitive secrecy rate and successful service ratio, thereby yielding the highest normalized composite utility.

The rest of this paper is organized as follows. Section 2 presents the system model. Section 3 formulates the MDP-based problem transformation. Section 4 develops the HC-SAC-based joint optimization algorithm. Section 5 provides simulation results and performance analysis. Finally, conclusions are drawn in Section 6.

**Notation:** Bold uppercase letters, bold lowercase letters, and lowercase letters denote matrices, vectors, and scalars, respectively. ${\left(\cdot \right)}^{T}$ denotes the transpose operation, ∥·∥ denotes the Euclidean norm, E[·] denotes the expectation operator, and $j=\sqrt{-1}$ is the imaginary unit.

## 2. System Model

In the envisioned ISAC-enabled vehicular network, this paper focuses on secure and low-latency communication functionality. The sensing capability is treated as an auxiliary information source that can provide contextual information, such as vehicle distribution, blockage conditions, and mobility states, to the BS/UAV controller through the state acquisition mechanism discussed in Section 2.4. Accordingly, the following system model characterizes the communication-oriented optimization part of the ISAC-enabled network, while dedicated radar waveform design and sensing-performance optimization are beyond the scope of this work.

### 2.1. Geometry and Mobility Model

**Figure 1:**
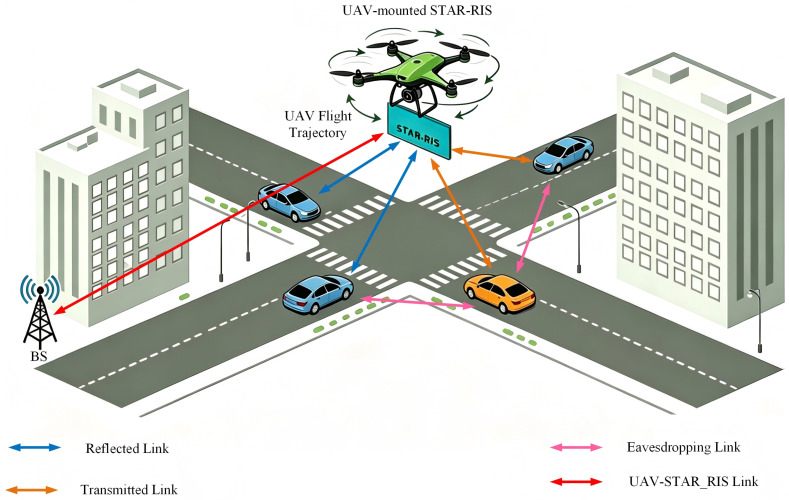
System model of secure and low-latency communications in UAV-mounted STAR-RIS-assisted urban vehicular networks.

Consider a typical urban road intersection scenario, where the system consists of one base station (BS), one UAV-mounted STAR-RIS, *K* legitimate vehicular users, and *E* passive eavesdropping vehicles, as shown in Figure 1.

The BS is deployed at a fixed roadside location to provide downlink communication services for the vehicles within the target area. Due to building blockage and high vehicle mobility, the direct BS-to-vehicle links may experience non-line-of-sight (NLoS) conditions for some users. To enhance the communication quality in blocked areas, a UAV carrying a STAR-RIS is deployed above the road to provide aerial assistance.

The total service duration is divided into *N* discrete time slots, each with a duration of $\delta_{t}$. Let the BS position be denoted by

(1) $$w_{b}={\left[x_{b},y_{b},0\right]}^{T}.$$

The UAV position at slot *n* is denoted by

(2) $$q\left[n\right]={\left[x\left[n\right],y\left[n\right],H\right]}^{T},\;n=1,2,\cdots ,N,$$

where *H* is the fixed UAV flight altitude. Therefore, the UAV trajectory optimization focuses on the two-dimensional horizontal deployment while maintaining a constant altitude, which is consistent with the rotary-wing propulsion energy model adopted in this paper. The position of the *k*-th legitimate vehicular user at slot *n* is given by

(3) $$u_{k}\left[n\right]={\left[x_{k}\left[n\right],y_{k}\left[n\right],0\right]}^{T},$$

and the position of the *e*-th passive eavesdropper at slot *n* is denoted by

(4) $$z_{e}\left[n\right]={\left[x_{e}\left[n\right],y_{e}\left[n\right],0\right]}^{T}.$$

Due to the UAV mobility limitation, the displacement between two adjacent slots must satisfy

(5) $$∥q\left[n+1\right]-q\left[n\right]∥\leq V_{\max}\delta_{t},\;n=1,\cdots ,N-1,$$

where $V_{\max}$ denotes the maximum UAV speed.

The STAR-RIS mounted on the UAV consists of *M* programmable elements. Owing to its simultaneous transmission and reflection capability, the reflection ratio and transmission ratio at slot *n* are denoted by $\beta_{r}\left[n\right]$ and $\beta_{t}\left[n\right]$, respectively, and satisfy

(6) $$\beta_{r}\left[n\right]+\beta_{t}\left[n\right]=1,\;0\leq \beta_{r}\left[n\right],\beta_{t}\left[n\right]\leq 1.$$

The corresponding reflection and transmission phase-shift matrices are defined as

(7) $$\Theta_{r}\left[n\right]=\mathrm{diag}\left(\sqrt{\beta_{r}\left[n\right]}e^{j\theta_{r,1}\left[n\right]},\cdots ,\sqrt{\beta_{r}\left[n\right]}e^{j\theta_{r,M}\left[n\right]}\right),$$

(8) $$\Theta_{t}\left[n\right]=\mathrm{diag}\left(\sqrt{\beta_{t}\left[n\right]}e^{j\theta_{t,1}\left[n\right]},\cdots ,\sqrt{\beta_{t}\left[n\right]}e^{j\theta_{t,M}\left[n\right]}\right),$$

where $\theta_{r,m}\left[n\right]$ and $\theta_{t,m}\left[n\right]$ denote the phase shifts of the *m*-th STAR-RIS element under the reflection and transmission modes, respectively.

### 2.2. Channel Gain Model

To characterize the urban blockage effect and small-scale fading, the channel between nodes *i* and *j* at slot *n* is modeled as

(9) $$h_{i,j}\left[n\right]=\sqrt{\rho_{0}{\left(d_{i,j}\left[n\right]\right)}^{-\alpha_{i,j}}}\left(\sqrt{\frac{\kappa_{i,j}}{1+\kappa_{i,j}}}\;h_{i,j}^{\mathrm{LoS}}\left[n\right]+\sqrt{\frac{1}{1+\kappa_{i,j}}}\;h_{i,j}^{\mathrm{NLoS}}\left[n\right]\right),$$

where $\rho_{0}$ denotes the channel power gain at the reference distance of 1 m, $d_{i,j}\left[n\right]$ is the Euclidean distance between nodes *i* and *j*, $\alpha_{i,j}$ is the path-loss exponent, and $\kappa_{i,j}$ denotes the Rician factor. Moreover, $h_{i,j}^{\mathrm{LoS}}\left[n\right]$ and $h_{i,j}^{\mathrm{NLoS}}\left[n\right]$ denote the deterministic LoS component and the random NLoS component, respectively.

For the BS–STAR-RIS link and the STAR-RIS–vehicle/eavesdropper links, which are mainly dominated by line-of-sight propagation, relatively large Rician factors are adopted. By contrast, for the direct BS-to-vehicle and BS-to-eavesdropper links in blocked urban environments, smaller Rician factors and larger path-loss exponents are used to characterize severe NLoS propagation conditions. Accordingly, the BS–STAR-RIS channel, the STAR-RIS–user channel, the STAR-RIS–eavesdropper channel, and the corresponding direct links are written, respectively, as

(10) $$h_{br}\left[n\right]=\sqrt{\rho_{0}{\left(d_{br}\left[n\right]\right)}^{-\alpha_{br}}}\left(\sqrt{\frac{\kappa_{br}}{1+\kappa_{br}}}\;h_{br}^{\mathrm{LoS}}\left[n\right]+\sqrt{\frac{1}{1+\kappa_{br}}}\;h_{br}^{\mathrm{NLoS}}\left[n\right]\right),$$

(11) $$g_{k}\left[n\right]=\sqrt{\rho_{0}{\left(d_{rk}\left[n\right]\right)}^{-\alpha_{rk}}}\left(\sqrt{\frac{\kappa_{rk}}{1+\kappa_{rk}}}\;g_{k}^{\mathrm{LoS}}\left[n\right]+\sqrt{\frac{1}{1+\kappa_{rk}}}\;g_{k}^{\mathrm{NLoS}}\left[n\right]\right),$$

(12) $$g_{e}\left[n\right]=\sqrt{\rho_{0}{\left(d_{re}\left[n\right]\right)}^{-\alpha_{re}}}\left(\sqrt{\frac{\kappa_{re}}{1+\kappa_{re}}}\;g_{e}^{\mathrm{LoS}}\left[n\right]+\sqrt{\frac{1}{1+\kappa_{re}}}\;g_{e}^{\mathrm{NLoS}}\left[n\right]\right),$$

(13) $$h_{d,k}\left[n\right]=\sqrt{\rho_{0}{\left(d_{bk}\left[n\right]\right)}^{-\alpha_{bk}}}\left(\sqrt{\frac{\kappa_{bk}}{1+\kappa_{bk}}}\;h_{d,k}^{\mathrm{LoS}}\left[n\right]+\sqrt{\frac{1}{1+\kappa_{bk}}}\;h_{d,k}^{\mathrm{NLoS}}\left[n\right]\right),$$

(14) $$h_{d,e}\left[n\right]=\sqrt{\rho_{0}{\left(d_{be}\left[n\right]\right)}^{-\alpha_{be}}}\left(\sqrt{\frac{\kappa_{be}}{1+\kappa_{be}}}\;h_{d,e}^{\mathrm{LoS}}\left[n\right]+\sqrt{\frac{1}{1+\kappa_{be}}}\;h_{d,e}^{\mathrm{NLoS}}\left[n\right]\right).$$

### 2.3. Imperfect CSI and Practical STAR-RIS Constraints

In the above channel model, the CSI is assumed to be available at the BS/UAV controller for resource optimization. However, in practical UAV-mounted STAR-RIS-assisted vehicular networks, channel estimation errors, phase quantization, and hardware impairments may exist due to vehicle mobility, limited pilot overhead, and finite-resolution STAR-RIS control circuits. To improve the practical interpretability of the proposed framework, this subsection discusses imperfect CSI and practical STAR-RIS constraints.

For the estimated channel coefficient, an additive CSI error model is adopted:

(15) $${\hat{h}}_{i,j}\left[n\right]=h_{i,j}\left[n\right]+e_{i,j}\left[n\right],$$

where $h_{i,j}\left[n\right]$ denotes the actual channel coefficient between nodes *i* and *j*, ${\hat{h}}_{i,j}\left[n\right]$ is the estimated CSI available at the controller, and $e_{i,j}\left[n\right]$ denotes the channel estimation error. The estimation error is modeled as a complex Gaussian random variable:

(16) $$e_{i,j}\left[n\right]∼\mathrm{CN}\left(0,\sigma_{e}^{2}\right),$$

where $\sigma_{e}^{2}$ characterizes the CSI uncertainty level. During policy execution, the HC-SAC agent makes decisions based on the estimated CSI ${\hat{h}}_{i,j}\left[n\right]$, while the actual received signal quality is affected by the true channel $h_{i,j}\left[n\right]$.

Moreover, practical STAR-RIS elements usually support only finite-resolution phase shifts. Therefore, the continuous phase shift $\theta_{m}\left[n\right]$ can be quantized into a finite codebook:

(17) $$F_{B}=\left\{0,\frac{2\pi }{2^{B}},\ldots ,\frac{2\pi (2^{B}-1)}{2^{B}}\right\},$$

where *B* denotes the number of phase quantization bits. The quantized phase shift is given by

(18) $$\theta_{m}^{Q}\left[n\right]=\arg\min_{\theta \in F_{B}}\left|\theta -\theta_{m}\left[n\right]\right|.$$

Accordingly, the practical STAR-RIS phase-shift matrices can be written as

(19) $$\Theta_{r}^{Q}\left[n\right]=\mathrm{diag}\left(e^{j\theta_{r,1}^{Q}\left[n\right]},\ldots ,e^{j\theta_{r,M}^{Q}\left[n\right]}\right),$$

(20) $$\Theta_{t}^{Q}\left[n\right]=\mathrm{diag}\left(e^{j\theta_{t,1}^{Q}\left[n\right]},\ldots ,e^{j\theta_{t,M}^{Q}\left[n\right]}\right).$$

In addition, STAR-RIS hardware impairments can be modeled by amplitude attenuation factors. Specifically, the practical reflection and transmission coefficients are expressed as

(21) $${\tilde{\beta }}_{r,m}\left[n\right]=\eta_{r}\beta_{r,m}\left[n\right],\;{\tilde{\beta }}_{t,m}\left[n\right]=\eta_{t}\beta_{t,m}\left[n\right],$$

where $0<\eta_{r}\leq 1$ and $0<\eta_{t}\leq 1$ denote the hardware efficiency factors of the reflection and transmission modes, respectively. When $\eta_{r}=\eta_{t}=1$ and B→∞, the ideal STAR-RIS model is recovered.

In the main algorithm design, the proposed HC-SAC framework is trained based on the estimated CSI and continuous STAR-RIS control variables. Imperfect CSI, finite-resolution phase control, and hardware impairments are included here as practical modeling considerations, and their impact is further evaluated in the robustness analysis in Section 5.10.

### 2.4. Vehicle State Acquisition and Control Signaling

In practical UAV-mounted STAR-RIS-assisted vehicular networks, the STAR-RIS does not independently detect vehicular users or estimate their channels. Instead, vehicle state acquisition and STAR-RIS control are coordinated by the BS and the UAV-mounted controller through vehicular signaling, pilot transmission, and sensing-assisted state estimation. To clarify the practical operation of the considered system, this subsection describes the vehicle state acquisition and control signaling mechanism.

Specifically, each vehicular user periodically broadcasts basic safety messages (BSMs) or cooperative awareness messages (CAMs), which contain information on the user’s position, velocity, moving direction, and service-related information. Meanwhile, uplink pilot signals are transmitted to assist the BS/UAV controller in estimating the channel state information (CSI). In sensing-assisted vehicular networks, onboard or roadside sensing functions can further provide auxiliary information on vehicle distribution, blockage conditions, and potential target states. Based on these signaling and sensing results, the BS or UAV-mounted controller constructs the system state, including vehicle mobility information, queue states, channel-related features, and eavesdropper-related information.

After obtaining the system state at the beginning of each time slot, the proposed HC-SAC policy generates the UAV trajectory control, STAR-RIS transmission–reflection partition ratio, phase-shift configuration, and transmit power allocation. The resulting control commands are then delivered to the UAV flight controller and the STAR-RIS controller through a dedicated low-rate control link. The UAV adjusts its position according to the trajectory command, while the STAR-RIS updates its transmission–reflection coefficients and phase shifts according to the received configuration command.

For analytical tractability, the duration of each time slot is assumed to be sufficiently short such that the vehicle positions, channel states, and queue states remain approximately unchanged within one slot. At the beginning of the next time slot, the BS/UAV controller updates the observed system state according to newly received BSM/CAM messages, pilot measurements, and sensing feedback. Therefore, the proposed framework operates in a closed-loop manner, where vehicle state acquisition, policy decision, STAR-RIS control, and environment update are repeatedly performed over time.

### 2.5. Secure Communication Model

Assume that the BS employs downlink superposition transmission at slot *n*, and the transmitted signal is expressed as

(22) $$x\left[n\right]=\sum_{k=1}^{K}\sqrt{p_{k}\left[n\right]}s_{k}\left[n\right],$$

where $p_{k}\left[n\right]$ denotes the transmit power allocated to the *k*-th legitimate user, and $s_{k}\left[n\right]$ is the corresponding information symbol satisfying $E[|s_{k}\left[n\right]{|}^{2}]=1$. The total transmit power is constrained by

(23) $$\sum_{k=1}^{K}p_{k}\left[n\right]\leq P_{\max},\;\forall n.$$

Since vehicular users are located on both sides of the road, different users may be served by either the reflection region or the transmission region of the STAR-RIS. Let $o_{k}\left[n\right]\in \left\{r,t\right\}$ denote the operating mode associated with user *k* at slot *n*, where *r* and *t* represent the reflection and transmission modes, respectively. Then, the equivalent channel from the BS to the *k*-th legitimate user can be written as

(24) $$h_{k}^{\mathrm{eq}}\left[n\right]=h_{d,k}\left[n\right]+g_{k}^{H}\left[n\right]\Theta_{o_{k}\left[n\right]}\left[n\right]h_{br}\left[n\right].$$

Accordingly, the received signal-to-interference-plus-noise ratio (SINR) of user *k* at slot *n* is given by

(25) $$\gamma_{k}\left[n\right]=\frac{p_{k}\left[n\right]{\left|h_{k}^{\mathrm{eq}}\left[n\right]\right|}^{2}}{\sum_{i\neq k}p_{i}\left[n\right]{\left|h_{k}^{\mathrm{eq}}\left[n\right]\right|}^{2}+\sigma_{k}^{2}},$$

where $\sigma_{k}^{2}$ denotes the receiver noise power. The achievable rate of user *k* is thus

(26) $$R_{k}\left[n\right]=B\log_{2}\left(1+\gamma_{k}\left[n\right]\right),$$

where *B* denotes the system bandwidth.

For the *e*-th passive eavesdropper, the equivalent channel for intercepting the signal intended for user *k* is expressed as

(27) $$h_{e,k}^{\mathrm{eq}}\left[n\right]=h_{d,e}\left[n\right]+g_{e}^{H}\left[n\right]\Theta_{o_{k}\left[n\right]}\left[n\right]h_{br}\left[n\right].$$

The corresponding eavesdropping SINR is given by

(28) $$\gamma_{e,k}\left[n\right]=\frac{p_{k}\left[n\right]{\left|h_{e,k}^{\mathrm{eq}}\left[n\right]\right|}^{2}}{\sum_{i\neq k}p_{i}\left[n\right]{\left|h_{e,k}^{\mathrm{eq}}\left[n\right]\right|}^{2}+\sigma_{e}^{2}},$$

where $\sigma_{e}^{2}$ is the noise power at the eavesdropper. The corresponding eavesdropping rate is

(29) $$R_{e,k}\left[n\right]=B\log_{2}\left(1+\gamma_{e,k}\left[n\right]\right).$$

Considering the strongest eavesdropping threat, the instantaneous secrecy rate of user *k* at slot *n* is defined as

(30) $$R_{k}^{\sec}\left[n\right]={\left[R_{k}\left[n\right]-\max_{e\in \{1,\cdots ,E\}}R_{e,k}\left[n\right]\right]}^{+},$$

where ${\left[x\right]}^{+}=\max\left(x,0\right)$.

### 2.6. Queue Evolution and Low-Latency Service Model

**Figure 2:**
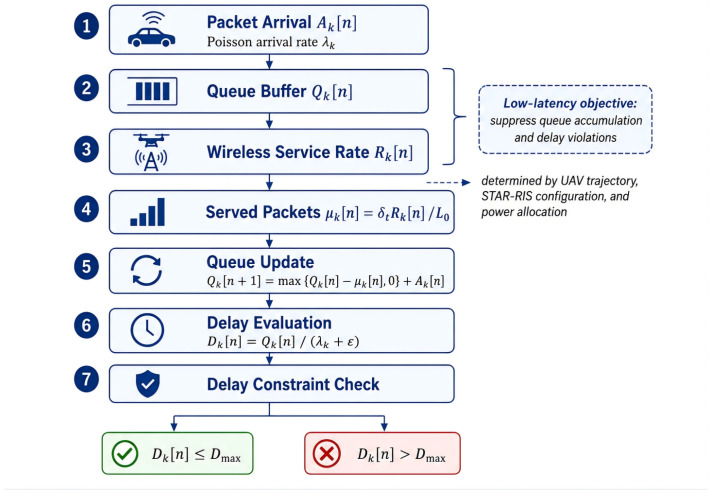
Queue evolution and a low-latency service model for vehicular users.

In urban vehicular networks, many safety-related services, such as cooperative driving, road hazard warning, and vehicle status reporting, are delay-sensitive. Therefore, in addition to improving the secrecy performance, it is necessary to explicitly characterize the traffic queue evolution and the corresponding service delay. Figure 2 illustrates the queue evolution and low-latency service model for each vehicular user.

Let $A_{k}\left[n\right]$ denote the newly arrived data packets of the *k*-th vehicular user at time slot *n*. The packet arrival process is modeled as a Poisson process with average arrival rate $\lambda_{k}$, i.e.,

(31) $$A_{k}\left[n\right]∼\mathrm{Poisson}\left(\lambda_{k}\right),$$

where $\lambda_{k}$ denotes the average packet arrival rate of user *k*. Let $Q_{k}\left[n\right]$ denote the queue length of the *k*-th vehicular user at the beginning of time slot *n*. The service capability of the system depends on the achievable communication rate. Given the achievable rate $R_{k}\left[n\right]$, the slot duration $\delta_{t}$, and the packet size $L_{0}$ in bits, the number of packets that can be served during time slot *n* is expressed as

(32) $$\mu_{k}\left[n\right]=\frac{\delta_{t}R_{k}\left[n\right]}{L_{0}},$$

where $\delta_{t}$ denotes the duration of one time slot and $L_{0}$ denotes the packet size. In the optimization formulation, $\mu_{k}\left[n\right]$ is treated as a continuous service amount to facilitate differentiable and low-complexity policy learning. In practical packet-level implementation, the actual number of served packets can be obtained by integer rounding, e.g., $\left\lfloor \mu_{k}\left[n\right]\right\rfloor$.

Accordingly, the queue evolution of vehicular user *k* is given by

(33) $$Q_{k}\left[n+1\right]=\max\left\{Q_{k}\left[n\right]-\mu_{k}\left[n\right],0\right\}+A_{k}\left[n\right].$$

This equation indicates that the queue length in the next slot is jointly determined by the remaining packets after service and the newly arrived packets.

Based on Little’s law, the instantaneous queueing delay of the *k*-th vehicular user can be approximated by the ratio between the queue length and the packet arrival rate, i.e.,

(34) $$D_{k}\left[n\right]=\frac{Q_{k}\left[n\right]}{\lambda_{k}+\epsilon },$$

where ϵ is a small positive constant used to avoid division by zero. This instantaneous delay approximation based on Little’s law has been widely adopted in cross-layer wireless resource optimization, since it provides a low-complexity delay feedback indicator for dynamic control problems. The average system delay at time slot *n* is then defined as

(35) $$D\left[n\right]=\frac{1}{K}\sum_{k=1}^{K}D_{k}\left[n\right],$$

where *K* denotes the number of vehicular users.

To capture the low-latency service requirement, a delay violation indicator is defined as

(36) $$I_{k}^{D}\left[n\right]=\left\{\begin{array}{cc} 1, & D_{k}\left[n\right]>D_{\max},\\ 0, & D_{k}\left[n\right]\leq D_{\max}, \end{array}\right.$$

where $D_{\max}$ is the maximum tolerable delay threshold. This queue-aware delay model is incorporated into the reward function of the proposed DRL framework, so that the learned control policy can jointly improve secrecy performance and suppress queue accumulation.

### 2.7. UAV Flight Energy Consumption Model

Since the UAV-mounted STAR-RIS needs to continuously adjust its aerial position to assist vehicular users in dynamic urban environments, UAV propulsion energy consumption should be explicitly considered. Instead of using a simplified quadratic displacement-based energy model, this paper adopts a practical rotary-wing UAV propulsion power model, which is widely used to characterize the relationship between UAV flight speed and propulsion energy consumption.

Let $q\left[n\right]={\left[x_{u}\left[n\right],y_{u}\left[n\right],H\right]}^{T}$ denote the UAV position at time slot *n*, where *H* is the fixed flight altitude. The horizontal flight speed of the UAV during time slot *n* is given by

(37) $$v\left[n\right]=\frac{∥q[n+1]-q[n]∥}{\delta_{t}},$$

where $\delta_{t}$ denotes the duration of one time slot. The propulsion power consumption of the rotary-wing UAV is modeled as

(38) $$P_{\mathrm{UAV}}\left(v\left[n\right]\right)=P_{0}\left(1+\frac{3v^{2}\left[n\right]}{U_{\mathrm{tip}}^{2}}\right)+P_{i}{\left(\sqrt{1+\frac{v^{4}\left[n\right]}{4v_{0}^{4}}}-\frac{v^{2}\left[n\right]}{2v_{0}^{2}}\right)}^{\frac{1}{2}}+\frac{1}{2}d_{0}\rho sAv^{3}\left[n\right],$$

where $P_{0}$ and $P_{i}$ denote the blade profile power and induced power in hovering status, respectively; $U_{\mathrm{tip}}$ is the tip speed of the rotor blade; $v_{0}$ is the mean rotor induced velocity in hovering; $d_{0}$ is the fuselage drag ratio; ρ is the air density; *s* is the rotor solidity; and *A* is the rotor disc area.

Accordingly, the UAV propulsion energy consumption during time slot *n* is expressed as

(39) $$E_{\mathrm{UAV}}\left[n\right]=P_{\mathrm{UAV}}\left(v\left[n\right]\right)\delta_{t}.$$

The total UAV propulsion energy consumption over the whole flight period is given by

(40) $$E_{\mathrm{tot}}^{\mathrm{UAV}}=\sum_{n=1}^{N}E_{\mathrm{UAV}}\left[n\right].$$

Recall that the UAV trajectory should satisfy the mobility constraint and the total propulsion energy budget:

(41) $$∥q[n+1]-q[n]∥\leq V_{\max}\delta_{t},\;\forall n,$$

(42) $$E_{\mathrm{tot}}^{\mathrm{UAV}}\leq E_{\max},$$

where $V_{\max}$ denotes the maximum UAV speed and $E_{\max}$ denotes the available UAV energy budget.

## 3. MDP Modeling and Problem Transformation

### 3.1. MDP Modeling

The considered joint optimization problem is high-dimensional, non-convex, strongly coupled, and long-term dynamic. Since vehicle positions, traffic arrivals, eavesdropping threats, and channel states all evolve over time, the control action taken at the current time slot affects not only the instantaneous secrecy rate and delay performance, but also the future system performance through UAV movement and queue evolution. Therefore, the original problem is modeled as a Markov decision process (MDP) with continuous state and action spaces.

At time slot *n*, the agent observes the system state s[n], executes an action a[n], and receives an immediate reward r[n]. The objective is to learn a policy π that maximizes the long-term expected discounted reward:

(43) $$\max_{\pi }\;E_{\pi }\left[\sum_{n=1}^{N}\zeta^{\;n-1}r\left[n\right]\right],$$

where ζ∈(0,1) is the discount factor.

### 3.2. State Space Design

To enable the agent to fully perceive the current communication environment, queue states, and UAV operating conditions, the system state is defined as

(44) $$s\left[n\right]=\left\{q[n],U[n],Z[n],Q[n],h[n],\beta [n-1],P[n-1]\right\},$$

where:

- q[n] denotes the current UAV position;
- $U\left[n\right]={\left\{u_{k}\left[n\right]\right\}}_{k=1}^{K}$ denotes the set of legitimate user positions;
- $Z\left[n\right]={\left\{z_{e}\left[n\right]\right\}}_{e=1}^{E}$ denotes the set of eavesdropper positions;
- $Q\left[n\right]={\left\{Q_{k}\left[n\right]\right\}}_{k=1}^{K}$ denotes the traffic queue states;
- h[n] represents the channel-state features composed of the BS–STAR-RIS, STAR-RIS–user, and STAR-RIS–eavesdropper links;
- β[n−1] and P[n−1] denote the STAR-RIS partition ratio and power allocation of the previous slot, respectively.

The above state representation jointly reflects spatial geometry, channel conditions, eavesdropping threats, and queue states. In addition, the previous-slot STAR-RIS partition ratio and power allocation are included to provide temporal context for consecutive decisions.

### 3.3. Action Space Design

At each time slot, the agent needs to jointly control the UAV trajectory, STAR-RIS transmission–reflection partition ratio, phase-shift configuration, and power allocation. Therefore, the action is defined as

(45) $$a\left[n\right]=\left\{\Delta q\left[n\right],\beta_{r}\left[n\right],\theta_{r}\left[n\right],\theta_{t}\left[n\right],p\left[n\right]\right\},$$

where:

- Δq[n] denotes the UAV displacement control at slot *n*;
- $\beta_{r}\left[n\right]$ denotes the STAR-RIS reflection ratio, while the transmission ratio is obtained as $\beta_{t}\left[n\right]=1-\beta_{r}\left[n\right]$;
- $\theta_{r}\left[n\right]=\left[\theta_{r,1}\left[n\right],\cdots ,\theta_{r,M}\left[n\right]\right]$ is the reflection phase-shift vector;
- $\theta_{t}\left[n\right]=\left[\theta_{t,1}\left[n\right],\cdots ,\theta_{t,M}\left[n\right]\right]$ is the transmission phase-shift vector;
- $p\left[n\right]=[p_{1}\left[n\right],\cdots ,p_{K}\left[n\right]]$ is the power allocation vector.

### 3.4. Reward Function Design

To jointly improve secure communication, low-latency performance, and service reliability while controlling the UAV mobility cost, the immediate reward at slot *n* is designed as

(46) $$r\left[n\right]=\alpha R_{\sec}\left[n\right]-\beta D\left[n\right]+\gamma C\left[n\right]-\lambda E_{\mathrm{UAV}}\left[n\right]-\xi \Phi \left[n\right],$$

where

(47) $$R_{\sec}\left[n\right]=\frac{1}{K}\sum_{k=1}^{K}R_{k}^{\sec}\left[n\right]$$

is the average secrecy rate,

(48) $$C\left[n\right]=\frac{1}{K}\sum_{k=1}^{K}I\left(R_{k}\left[n\right]\geq R_{k}^{\min}\right)$$

is the successful service ratio, $E_{\mathrm{UAV}}\left[n\right]$ denotes the UAV propulsion energy consumption in slot *n*, and Φ[n] denotes the degree of constraint violation. The weighting factors α, β, γ, λ, and ξ are nonnegative constants.

## 4. HC-SAC-Based Joint Optimization Algorithm

### 4.1. HC-SAC Framework Overview

**Figure 3:**
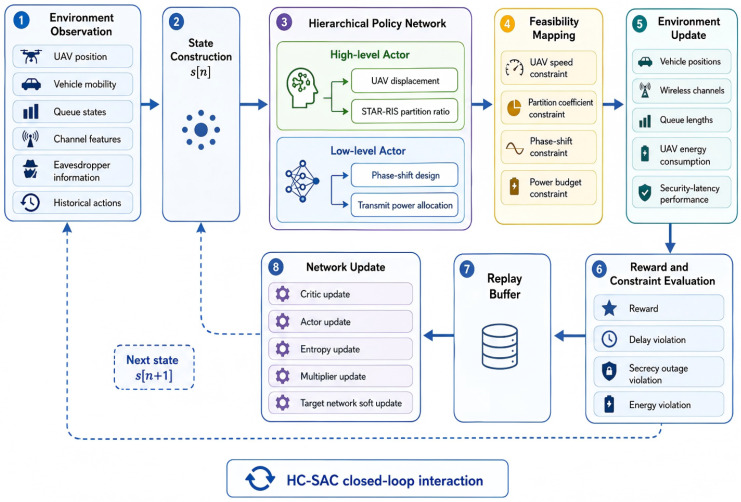
Step-by-step methodology of the proposed HC-SAC-based joint optimization framework.

As illustrated in Figure 3, the proposed HC-SAC framework follows a closed-loop interaction process between the learning agent and the UAV-mounted STAR-RIS-assisted vehicular environment. At each time slot, the current network state is constructed from the UAV position, vehicle mobility information, queue states, channel-related features, eavesdropper information, and historical control actions. Based on this state, the hierarchical policy network generates continuous control actions for UAV movement, STAR-RIS transmission–reflection configuration, phase-shift design, and transmit power allocation.

Before being executed in the environment, the raw actions are transformed by a feasibility mapping module to satisfy the UAV mobility constraint, STAR-RIS coefficient constraint, phase-shift constraint, and power budget constraint. The environment then updates the vehicle positions, wireless channels, queue lengths, UAV energy consumption, and security–latency performance. The resulting reward and constraint violation signals are stored in the replay buffer and used to update the actor networks, critic networks, entropy coefficient, and constraint multipliers. The detailed definitions of the hierarchical policy structure, feasibility mapping, reward shaping, and network updates are provided in the following subsections.

### 4.2. Hierarchical Constrained SAC Update

Different from standard SAC, the proposed HC-SAC introduces a hierarchical control structure and a constraint-aware reward shaping mechanism. The high-level actor is responsible for coarse-grained control variables, including the UAV displacement and the STAR-RIS transmission–reflection partition ratio, while the low-level actor is responsible for fine-grained physical-layer decisions, including the STAR-RIS phase-shift vectors and transmit power allocation. This decomposition reduces the effective action coupling and improves learning stability in dynamic vehicular environments.

Let $a_{h}\left[n\right]$ and $a_{l}\left[n\right]$ denote the high-level and low-level actions at time slot *n*, respectively. The overall action is given by

(49) $$a\left[n\right]=\{a_{h}\left[n\right],a_{l}\left[n\right]\}.$$

The joint hierarchical policy can be expressed as

(50) $$\pi_{\phi }\left(a\left[n\right]|s\left[n\right]\right)=\pi_{\phi_{h}}\left(a_{h}\left[n\right]|s\left[n\right]\right)\pi_{\phi_{l}}\left(a_{l}\left[n\right]|s\left[n\right],a_{h}\left[n\right]\right),$$

where $\phi_{h}$ and $\phi_{l}$ denote the parameters of the high-level and low-level actors, respectively, and $\phi =\{\phi_{h},\phi_{l}\}$.

Two critic networks with parameters $\omega_{1}$ and $\omega_{2}$ are introduced to estimate the soft action-value functions $Q_{\omega_{1}}\left(s,a\right)$ and $Q_{\omega_{2}}\left(s,a\right)$, respectively. In addition, two target critic networks with parameters ${\bar{\omega }}_{1}$ and ${\bar{\omega }}_{2}$ are maintained to improve the stability of temporal-difference learning.

The entropy-regularized objective of the hierarchical policy is given by

(51) $$J\left(\pi \right)=\sum_{n=1}^{N}E_{\left(s\left[n\right],a\left[n\right]\right)∼\rho_{\pi }}\left[r\left[n\right]+\tau H\left(\pi_{\phi }\left(\cdot |s\left[n\right]\right)\right)\right],$$

where τ is the entropy temperature parameter and $H(\pi_{\phi }\left(\cdot |s\left[n\right]\right))$ denotes the policy entropy.

To explicitly account for delay, secrecy outage, and UAV energy-related constraints, a Lagrangian-style reward shaping mechanism is incorporated into the learning process. Let $\psi_{d}$, $\psi_{s}$, and $\psi_{e}$ denote the adaptive multipliers associated with delay violation, secrecy outage violation, and UAV energy violation, respectively. The corresponding violation levels are defined as

(52) $$\Delta_{d}\left[n\right]={\left[D\left[n\right]-D_{\max}\right]}^{+},$$

(53) $$\Delta_{s}\left[n\right]={\left[P_{\mathrm{out}}\left[n\right]-P_{\mathrm{out}}^{\max}\right]}^{+},$$

To handle the total UAV energy budget in an online learning process, the accumulated UAV propulsion energy up to slot *n* is defined as

(54) $${\bar{E}}_{\mathrm{UAV}}\left[n\right]=\sum_{i=1}^{n}E_{\mathrm{UAV}}\left[i\right].$$

The corresponding energy violation level is defined as

(55) $$\Delta_{e}\left[n\right]={\left[{\bar{E}}_{\mathrm{UAV}}\left[n\right]-\frac{n}{N}E_{\max}\right]}^{+},$$

where ${\left[x\right]}^{+}=\max\left\{x,0\right\}$, $D_{\max}$ is the maximum tolerable delay threshold, $P_{\mathrm{out}}^{\max}$ is the maximum tolerable secrecy outage probability, and $E_{\max}$ is the total UAV energy budget.

The constraint-aware shaped reward is then expressed as

(56) $$\hat{r}\left[n\right]=r\left[n\right]-\psi_{d}\Delta_{d}\left[n\right]-\psi_{s}\Delta_{s}\left[n\right]-\psi_{e}\Delta_{e}\left[n\right].$$

During each training iteration, the target value for the critic networks is constructed as

(57) $$y\left[n\right]=\hat{r}\left[n\right]+\zeta \left(\min_{j=1,2}Q_{{\bar{\omega }}_{j}}\left(s\left[n+1\right],a\left[n+1\right]\right)-\tau \log\pi_{\phi }\left(a\left[n+1\right]|s\left[n+1\right]\right)\right),$$

where ζ is the discount factor and $a\left[n+1\right]∼\pi_{\phi }\left(\cdot |s\left[n+1\right]\right)$. The critic networks are updated by minimizing the soft Bellman residual:

(58) $$L\left(\omega_{j}\right)=E_{(s,a,\hat{r},s^{\prime })∼D}\left[{\left(Q_{\omega_{j}}\left(s\left[n\right],a\left[n\right]\right)-y\left[n\right]\right)}^{2}\right],\;j=1,2,$$

where D denotes the replay buffer.

The hierarchical policy is updated by minimizing

(59) $$J\left(\phi \right)=E_{s\left[n\right]∼D,a\left[n\right]∼\pi_{\phi }}\left[\tau \log\pi_{\phi }\left(a\left[n\right]|s\left[n\right]\right)-\min_{j=1,2}Q_{\omega_{j}}\left(s\left[n\right],a\left[n\right]\right)\right].$$

To enhance exploration adaptivity, an automatic entropy tuning mechanism is adopted. The temperature parameter τ is updated by minimizing

(60) $$J\left(\tau \right)=E_{a\left[n\right]∼\pi_{\phi }}\left[-\tau \left(\log\pi_{\phi }\left(a\left[n\right]|s\left[n\right]\right)+\bar{H}\right)\right],$$

where $\bar{H}$ is the target entropy.

The constraint multipliers are updated according to the observed violation levels:

(61) $$\psi_{d}\leftarrow {\left[\psi_{d}+\eta_{\psi }\Delta_{d}\left[n\right]\right]}^{+},$$

(62) $$\psi_{s}\leftarrow {\left[\psi_{s}+\eta_{\psi }\Delta_{s}\left[n\right]\right]}^{+},$$

(63) $$\psi_{e}\leftarrow {\left[\psi_{e}+\eta_{\psi }\Delta_{e}\left[n\right]\right]}^{+},$$

where $\eta_{\psi }$ is the multiplier learning rate.

Finally, the target critic networks are softly updated as

(64) $${\bar{\omega }}_{j}\leftarrow \rho \omega_{j}+\left(1-\rho \right){\bar{\omega }}_{j},\;j=1,2,$$

where ρ∈(0,1) is the soft update coefficient.

### 4.3. Feasibility Mapping of Continuous Actions

Since the raw actions generated by the actor networks may violate physical constraints, a feasibility mapping operation is performed before interacting with the environment. Let the hierarchical actor output the raw action

(65) $$\tilde{a}\left[n\right]=\left\{\Delta \tilde{q}\left[n\right],{\tilde{\beta }}_{r}\left[n\right],{\tilde{\theta }}_{r}\left[n\right],{\tilde{\theta }}_{t}\left[n\right],\tilde{p}\left[n\right]\right\}.$$

For the UAV displacement, the mapped motion vector is given by

(66) $$\Delta q\left[n\right]=\min\left\{1,\frac{V_{\max}\delta_{t}}{∥\Delta \tilde{q}\left[n\right]∥+\epsilon }\right\}\Delta \tilde{q}\left[n\right],$$

where $V_{\max}$ is the maximum UAV speed, $\delta_{t}$ is the slot duration, and ϵ>0 is a small constant used to avoid division by zero. The UAV position is then updated as

(67) $$q\left[n+1\right]=\Pi_{Q}\left(q[n]+\Delta q[n]\right),$$

where $\Pi_{Q}\left(\cdot \right)$ denotes the projection onto the feasible flight region Q.

For the STAR-RIS transmission–reflection partition ratio, the raw output is mapped by a sigmoid function:

(68) $$\beta_{r}\left[n\right]=\frac{1}{1+\exp(-{\tilde{\beta }}_{r}\left[n\right])},$$

and the transmission ratio is given by

(69) $$\beta_{t}\left[n\right]=1-\beta_{r}\left[n\right].$$

Thus, the constraints $0\leq \beta_{r}\left[n\right]\leq 1$, $0\leq \beta_{t}\left[n\right]\leq 1$, and $\beta_{r}\left[n\right]+\beta_{t}\left[n\right]=1$ are satisfied.

For the STAR-RIS phase-shift vectors, a bounded mapping is applied:

(70) $$\theta_{r,m}\left[n\right]=\pi \left(\tanh({\tilde{\theta }}_{r,m}\left[n\right])+1\right),\;\forall m,$$

(71) $$\theta_{t,m}\left[n\right]=\pi \left(\tanh({\tilde{\theta }}_{t,m}\left[n\right])+1\right),\;\forall m.$$

Therefore, $\theta_{r,m}\left[n\right]\in \left[0,2\pi \right)$ and $\theta_{t,m}\left[n\right]\in \left[0,2\pi \right)$ are guaranteed.

For the transmit power allocation, a softmax-based normalization is adopted:

(72) $${\bar{p}}_{k}\left[n\right]=\frac{\exp({\tilde{p}}_{k}\left[n\right])}{\sum_{i=1}^{K}\exp\left({\tilde{p}}_{i}\left[n\right]\right)},\;\forall k.$$

The feasible transmit power is obtained as

(73) $$p_{k}\left[n\right]=P_{\max}{\bar{p}}_{k}\left[n\right],\;\forall k.$$

This mapping guarantees $p_{k}\left[n\right]\geq 0$ and $\sum_{k=1}^{K}p_{k}\left[n\right]=P_{\max}$. In this paper, the softmax mapping is used to allocate the available transmit power budget among vehicular users for secrecy-rate-oriented transmission.

### 4.4. Algorithm Procedure

The overall procedure of the proposed HC-SAC-based joint optimization algorithm is summarized in Algorithm 1.
**Algorithm 1** Proposed HC-SAC-based joint optimization algorithm  1:Initialize the vehicular environment, replay buffer D, high-level actor, low-level actor, critic networks, target critic networks, entropy coefficient, and constraint multipliers.  2:**for** each training episode **do**  3:    Reset the environment and obtain the initial state s[1].  4:    **for** each time slot n=1,…,N **do**  5:        Observe the current state s[n].  6:        Generate the raw action $\tilde{a}\left[n\right]$ from the hierarchical policy networks.  7:        Apply feasibility mapping to obtain the valid action a[n].  8:        Execute a[n] in the environment.  9:        Update the UAV position, vehicle positions, channel states, queue states, and UAV energy consumption.10:        Calculate the immediate reward r[n] and constraint violation levels $\Delta_{d}\left[n\right]$, $\Delta_{s}\left[n\right]$, and $\Delta_{e}\left[n\right]$.11:        Compute the shaped reward $\hat{r}\left[n\right]$.12:        Observe the next state s[n+1].13:        Store $\left(s\left[n\right],a\left[n\right],\hat{r}\left[n\right],s\left[n+1\right]\right)$ into the replay buffer D.14:        **if** the replay buffer size is larger than the mini-batch size **then**15:            Sample a mini-batch from D.16:            Update the critic networks by minimizing the soft Bellman residual.17:            Update the high-level and low-level actors using the entropy-regularized policy objective.18:            Update the entropy coefficient.19:            Update the constraint multipliers.20:            Softly update the target critic networks.21:        **end if**22:    **end for**23:**end for**24:Output the trained hierarchical policy.

### 4.5. Complexity and Convergence Discussion

Let $N_{\phi_{h}}$, $N_{\phi_{l}}$, and $N_{\omega }$ denote the number of trainable parameters in the high-level actor, low-level actor, and each critic network, respectively. For each mini-batch update with batch size $B_{s}$, the dominant computational complexity of the proposed HC-SAC algorithm mainly comes from forward and backward propagation through the hierarchical actor networks and the two critic networks, which can be approximated as

(74) $$O\left(B_{s}\left(N_{\phi_{h}}+N_{\phi_{l}}+2N_{\omega }\right)\right).$$

If the training process contains $E_{\mathrm{ep}}$ episodes and each episode consists of *N* time slots, the overall offline training complexity is given by

(75) $$O\left(E_{\mathrm{ep}}NB_{s}\left(N_{\phi_{h}}+N_{\phi_{l}}+2N_{\omega }\right)\right).$$

Although HC-SAC introduces hierarchical action generation and constraint-aware multiplier updates, these operations bring only a small additional computational cost compared with standard actor–critic training. After training, the online execution only requires one forward propagation of the high-level and low-level actor networks, whose complexity is

(76) $$O\left(N_{\phi_{h}}+N_{\phi_{l}}\right).$$

Therefore, the trained policy can directly output the UAV displacement, STAR-RIS transmission–reflection partition ratio, phase-shift vectors, and transmit power allocation without repeatedly solving the original non-convex optimization problem, making it suitable for online decision-making in dynamic vehicular environments.

To improve training stability, the proposed HC-SAC uses double critics, target critic networks, experience replay, entropy-regularized policy improvement, and adaptive constraint multipliers. Experience replay reuses historical transitions, the entropy term encourages exploration, and constraint-aware reward shaping penalizes delay violation, secrecy outage, and excessive UAV energy consumption. Due to neural network approximation and the non-convex problem structure, global optimality cannot be guaranteed. The convergence curves in Section 5 show that HC-SAC reaches a stable evaluation score after training in the considered setting.

## 5. Simulation Results and Discussion

### 5.1. Simulation Settings

We consider a typical urban road intersection scenario with an area size of 500 m×500 m. The BS is deployed at a fixed roadside location, while the UAV provides aerial assistance above the road. The total service period is divided into N=30 time slots, and the duration of each slot is $\delta_{t}=1\;s$. Legitimate vehicular users and eavesdropping vehicles move along predefined lanes. The vehicular users follow a lane-based random mobility model, while the eavesdroppers follow a random lane-following mobility model. The traffic arrival process is modeled as a Poisson process to characterize the stochastic nature of vehicular service arrivals. During training, all DRL algorithms are implemented with the same state space, action space, reward definition, replay setting, and neural network scale unless otherwise specified.

### 5.2. Simulation Setup and Evaluation Metrics

In this section, numerical simulations are conducted to evaluate the performance of the proposed HC-SAC-based joint optimization scheme. Unless otherwise specified, the main simulation parameters are listed in Table 2. An urban vehicular communication scenario is considered, where vehicular users and potential eavesdroppers are distributed along the road. The UAV-mounted STAR-RIS dynamically adjusts its trajectory and transmission–reflection configuration according to vehicle mobility, blockage conditions, queue states, and channel-related information.

The propulsion energy parameters of the rotary-wing UAV are given in Table 3. These parameters are used to evaluate the practical UAV propulsion energy consumption in the proposed framework.

To ensure reproducibility, the main training hyperparameters of the proposed HC-SAC algorithm and the DRL-based benchmark schemes are summarized in Table 4. Unless otherwise specified, all DRL-based schemes are trained under the same vehicular mobility traces, channel realizations, eavesdropper distributions, state information, action constraints, reward components, and the number of environment interactions.

For fair comparison, PPO and DDPG adopt the same state representation, action mapping rules, reward components, neural network scale, and simulation scenarios as the proposed HC-SAC. The same vehicle trajectories, blockage distributions, channel realizations, eavesdropper locations, and traffic arrival processes are used for all DRL-based schemes. Therefore, the performance difference mainly comes from the learning architecture and policy update mechanism rather than from different simulation conditions.

To clarify the calculation of the simulation results, the evaluation metrics used in this paper are defined as follows. The average secrecy rate is calculated by

(77) $${\bar{R}}_{\sec}=\frac{1}{NK}\sum_{n=1}^{N}\sum_{k=1}^{K}R_{k}^{\sec}\left[n\right],$$

where $R_{k}^{\sec}\left[n\right]$ denotes the instantaneous secrecy rate of vehicular user *k* at time slot *n*.

The secrecy outage probability (SOP) is defined as the probability that the instantaneous secrecy rate falls below a predefined secrecy threshold $R_{\mathrm{th}}$. In the simulations, it is estimated by Monte Carlo counting as

(78) $$P_{\mathrm{out}}=\frac{\sum_{n=1}^{N}\sum_{k=1}^{K}I\left(R_{k}^{\sec}\left[n\right]<R_{\mathrm{th}}\right)}{NK},$$

where I(·) is the indicator function.

The average delay is calculated based on the queue-aware delay model as

(79) $$\bar{D}=\frac{1}{NK}\sum_{n=1}^{N}\sum_{k=1}^{K}D_{k}\left[n\right],$$

where $D_{k}\left[n\right]$ denotes the queueing delay of user *k* at slot *n*.

The successful service ratio (SSR) is defined as the ratio of vehicular users that simultaneously satisfy the rate and delay requirements:

(80) $$\mathrm{SSR}=\frac{1}{NK}\sum_{n=1}^{N}\sum_{k=1}^{K}I\left(R_{k}\left[n\right]\geq R_{k}^{\min},D_{k}\left[n\right]\leq D_{\max}\right),$$

where $R_{k}^{\min}$ is the minimum required communication rate.

As an auxiliary per-slot indicator, the instantaneous normalized utility can be written as

(81) $$U_{\mathrm{ins}}=w_{r}{\tilde{R}}_{\sec}+w_{c}\tilde{\mathrm{SSR}}-w_{d}\tilde{D}-w_{o}{\tilde{P}}_{\mathrm{out}},$$

where ${\tilde{R}}_{\sec}$, $\tilde{\mathrm{SSR}}$, $\tilde{D}$, and ${\tilde{P}}_{\mathrm{out}}$ denote the normalized secrecy rate, successful service ratio, average delay, and secrecy outage probability, respectively. The coefficients $w_{r}$, $w_{c}$, $w_{d}$, and $w_{o}$ are nonnegative weights used to balance different performance metrics.

Each reported point is obtained by averaging over 24 independent evaluation episodes under the corresponding parameter setting. Since the main purpose is to compare the average performance trends, only the mean values are plotted in the figures.

### 5.3. Benchmark Schemes

To comprehensively evaluate the effectiveness of the proposed method, several representative DRL-based and structural benchmark schemes are considered. The detailed definitions of these schemes are summarized in Table 5.

For fairness, all benchmark schemes are evaluated under the same vehicular mobility traces, channel realizations, eavesdropper distributions, blockage conditions, packet arrival processes, and initial UAV locations. Unless a specific variable is intentionally fixed for benchmark evaluation, the same state information, action constraints, reward components, and feasibility mapping rules are adopted.

### 5.4. Training Behavior Comparison

**Figure 4:**
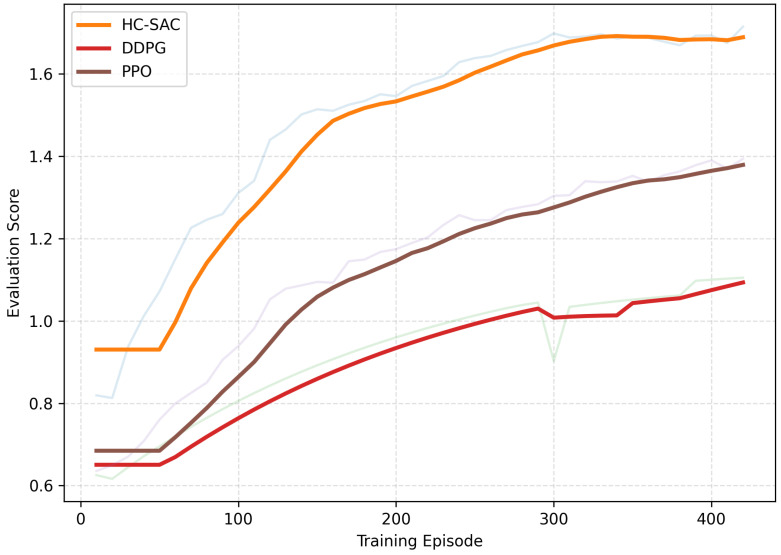
Convergence behavior of HC-SAC, DDPG, and PPO. Light-colored curves denote raw evaluation scores, and bold curves denote smoothed trends.

Figure 4 illustrates the convergence behavior of different DRL-based schemes in terms of evaluation score. It can be observed that the proposed HC-SAC achieves the highest evaluation score after convergence and exhibits a smooth upward trend throughout training. PPO also converges stably, but its final evaluation score remains below that of HC-SAC, whereas DDPG converges to the lowest level among the three DRL-based schemes. These results indicate that the hierarchical and constraint-aware design improves policy learning effectiveness in the considered dynamic vehicular environment. The convergence trend is also consistent with the final composite-utility ranking reported later.

### 5.5. Delay Performance Analysis

**Figure 5:**
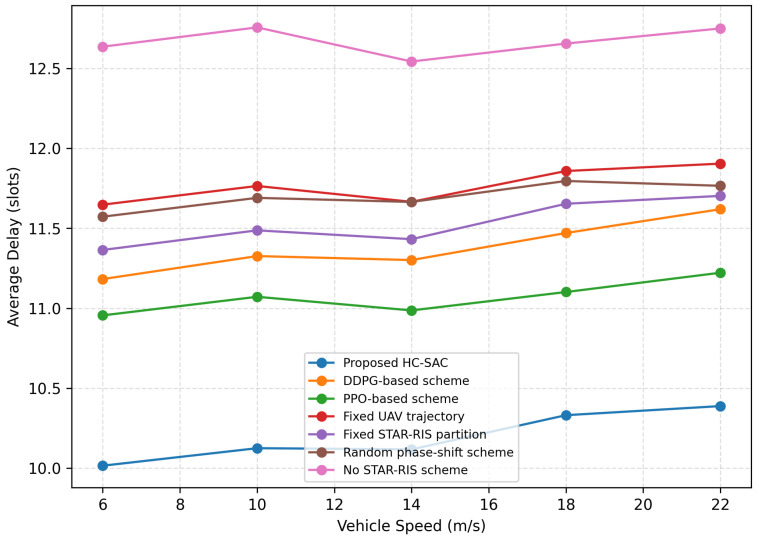
Average delay under different vehicle speeds.

Figure 5 shows the average delay under different vehicle speeds. As the vehicle speed increases, the average delay of all schemes generally rises due to faster topology variation and more severe channel fluctuation. Nevertheless, the proposed HC-SAC consistently achieves the lowest delay over the whole speed range, varying from 10.02 to 10.39 slots. By comparison, PPO varies from 10.96 to 11.22 slots, and DDPG varies from 11.18 to 11.62 slots. Averaged over the tested speed range, HC-SAC reduces delay by about 7.9% relative to PPO and 10.4% relative to DDPG. This result demonstrates that the hierarchical control structure and constraint-aware learning mechanism can better coordinate UAV mobility, STAR-RIS reconfiguration, and resource scheduling, thereby improving queue stability and delay control in highly dynamic vehicular environments.

Compared with all benchmark schemes, HC-SAC provides the strongest low-latency guarantee, while the delay gap with the structural baselines is even more evident.

### 5.6. Secure Communication Performance Analysis

**Figure 6:**
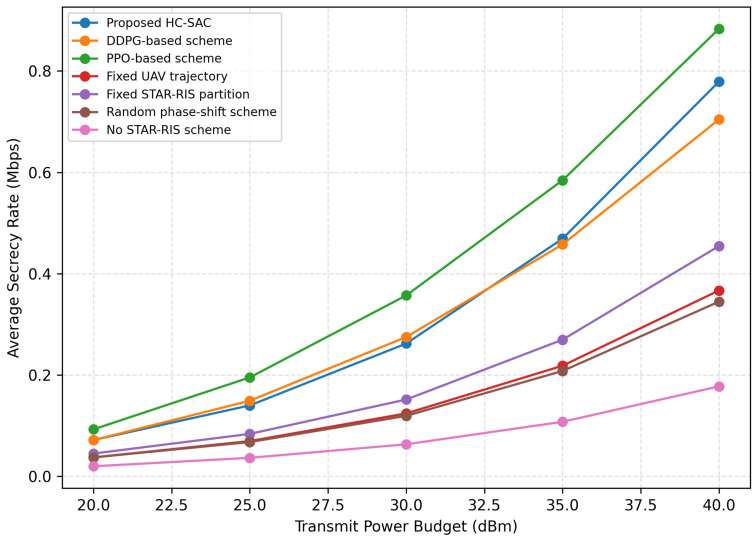
Average secrecy rate under different transmit power levels.

Figure 6 depicts the average secrecy rate under different BS transmit power budgets. As the transmit power increases, the secrecy rate of all schemes improves because stronger transmit power enhances the legitimate communication links. Among all DRL-based schemes, PPO achieves the highest secrecy rate, while the proposed HC-SAC provides competitive secrecy-rate performance and outperforms DDPG in the high-power regime. For example, at 40 dBm, PPO, HC-SAC, and DDPG achieve secrecy rates of 0.8832, 0.7791, and 0.7043 Mbps, respectively. In addition, all three DRL-based schemes outperform the structural baselines, suggesting the benefit of combining adaptive learning with UAV mobility and STAR-RIS-assisted propagation control.

Although HC-SAC does not achieve the highest secrecy rate, it still maintains a competitive secure transmission capability while simultaneously emphasizing delay and secrecy-reliability constraints.

**Figure 7:**
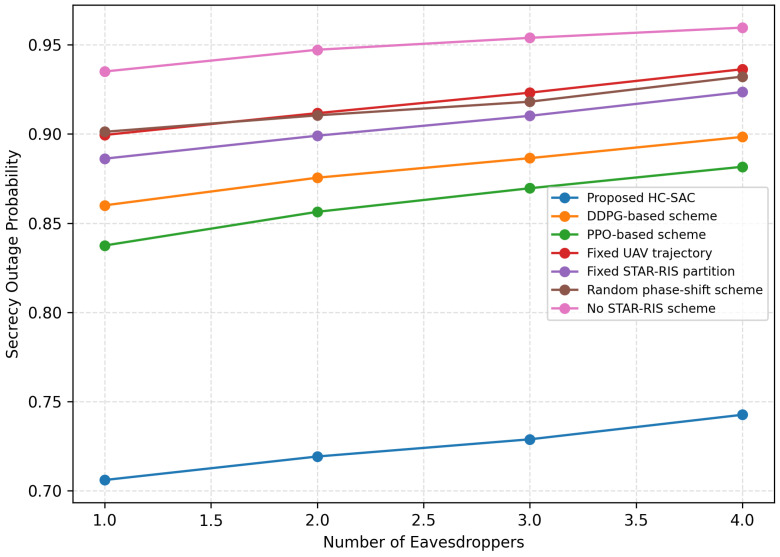
Secrecy outage probability versus the number of eavesdroppers.

Figure 7 illustrates the secrecy outage probability versus the number of eavesdroppers. As expected, the secrecy outage probability increases with the number of eavesdroppers for all schemes. However, the proposed HC-SAC consistently yields the lowest secrecy outage probability among all methods. Averaged over the tested eavesdropper settings, the SOP of HC-SAC is 0.7241, compared with 0.8613 for PPO and 0.8801 for DDPG, corresponding to reductions of about 15.9% and 17.7%, respectively. This result indicates that the proposed hierarchical constrained mechanism improves secrecy reliability under unfavorable wiretap conditions.

### 5.7. Service Reliability Analysis

**Figure 8:**
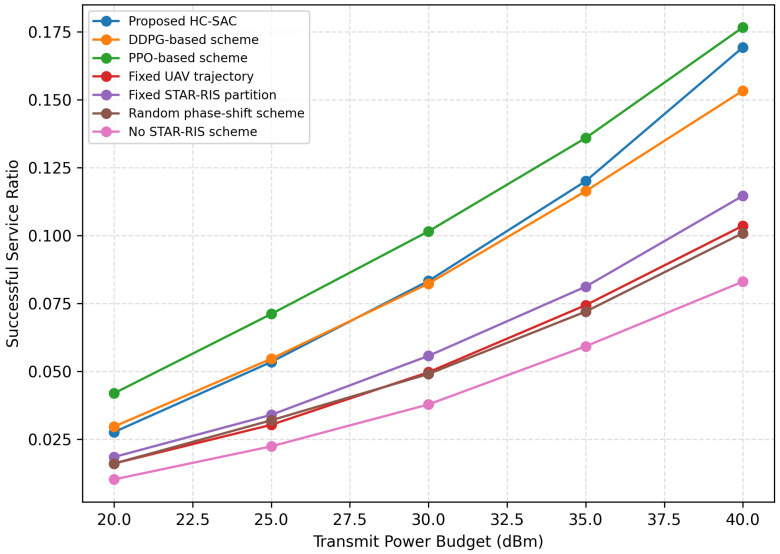
Successful service ratio under different transmit power levels.

Figure 8 presents the successful service ratio under different transmit power budgets, where the successful service ratio is defined as the proportion of users whose rate and delay requirements are simultaneously satisfied. It can be seen that the successful service ratio of all schemes increases with the transmit power budget. PPO achieves the highest successful service ratio, while the proposed HC-SAC remains close to PPO and becomes slightly better than DDPG in the medium- and high-power regimes. At 40 dBm, the SSR values of PPO, HC-SAC, and DDPG are 0.1767, 0.1693, and 0.1534, respectively.

These results show that HC-SAC preserves strong service capability while achieving better delay and secrecy outage performance. Therefore, compared with PPO, the proposed method provides a more balanced service-oriented control behavior rather than purely pursuing rate-oriented gains.

### 5.8. Composite Utility Analysis

To further evaluate the overall security–latency trade-off, a normalized composite utility is introduced based on the area-under-the-curve (AUC) values for the secrecy rate, successful service ratio, delay, and secrecy outage probability. Specifically, the secrecy rate and successful service ratio are positively normalized, whereas the delay and secrecy outage probability are inversely normalized. The resulting composite utility is defined as

(82) $$U_{\mathrm{comp}}=w_{1}{\tilde{\mathrm{AUC}}}_{R_{\sec}}+w_{2}{\tilde{\mathrm{AUC}}}_{\mathrm{SSR}}+w_{3}\left(1-{\tilde{\mathrm{AUC}}}_{D}\right)+w_{4}\left(1-{\tilde{\mathrm{AUC}}}_{\mathrm{SOP}}\right),$$

where ${\tilde{\mathrm{AUC}}}_{R_{\sec}}$, ${\tilde{\mathrm{AUC}}}_{\mathrm{SSR}}$, ${\tilde{\mathrm{AUC}}}_{D}$, and ${\tilde{\mathrm{AUC}}}_{\mathrm{SOP}}$ denote the normalized AUC values of secrecy rate, successful service ratio, delay, and secrecy outage probability, respectively. According to the objective of secure and low-latency vehicular communications, larger weights are assigned to delay and secrecy outage performance.

**Figure 9:**
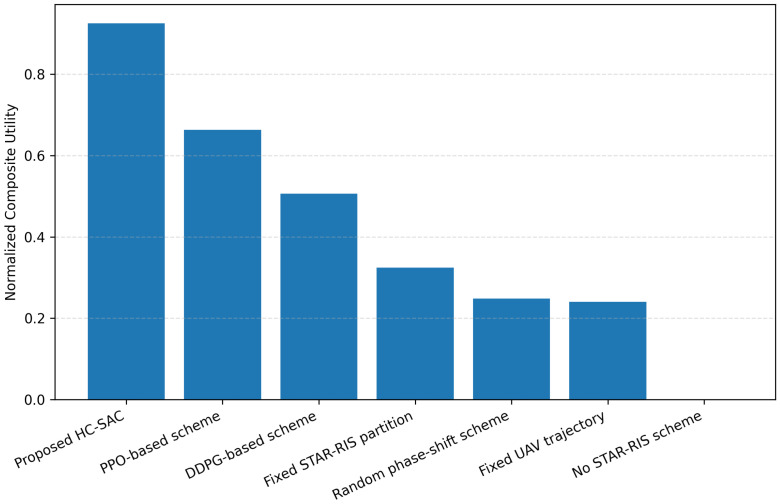
Normalized composite utility comparison of different schemes.

Figure 9 compares the normalized composite utility of all schemes. It can be observed that the proposed HC-SAC achieves the highest composite utility of 0.9254, while PPO, DDPG, the fixed STAR-RIS partition, the random phase-shift scheme, the fixed UAV trajectory scheme, and the no STAR-RIS scheme achieve 0.6630, 0.5060, 0.3241, 0.2480, 0.2403, and 0, respectively. The zero value of the No STAR-RIS scheme is caused by the min–max normalization, where the worst-performing scheme is mapped to zero. Therefore, HC-SAC improves the composite utility by about 39.6% over PPO and 82.9% over DDPG. Although PPO performs better in terms of average secrecy rate and successful service ratio, HC-SAC provides more significant advantages in delay and secrecy outage probability. As a result, the proposed method achieves the most balanced overall performance when security and latency are jointly considered. This ranking is mainly driven by the delay and SOP gains rather than rate maximization alone.

The above results also explain why the proposed HC-SAC achieves the highest normalized composite utility. Although PPO achieves a higher secrecy rate and successful service ratio in some cases, this does not necessarily indicate an overall advantage in the considered secure and low-latency vehicular communication scenario. PPO tends to learn a more rate-oriented policy, whereas the proposed HC-SAC adopts entropy-regularized off-policy learning and constraint-aware reward shaping to reduce queue accumulation and suppress secrecy outage events. Therefore, HC-SAC does not always maximize a single metric, but achieves a better overall security–latency trade-off, as reflected by its lower average delay, lower secrecy outage probability, and higher normalized composite utility.

### 5.9. Ablation Study

To further verify the contribution of the key components in the proposed framework, an ablation study is conducted by comparing four SAC-based variants under the same simulation environment, random seeds, and evaluation settings. The considered variants include standard SAC, HC-SAC without hierarchical control, HC-SAC without constraint-aware learning, and the complete proposed HC-SAC. The results averaged over three random seeds are reported in Table 6, where the secrecy rate is measured in Mbps, and the delay is measured in slots. Because the composite utility is normalized within this ablation group, it is used only for comparing the four SAC variants.

As shown in Table 6, the standard SAC baseline obtains the lowest composite utility, indicating that directly applying a flat SAC policy is insufficient for the considered high-dimensional joint control problem. When the hierarchical control structure is removed, the secrecy rate and successful service ratio remain relatively high, but the delay and secrecy outage probability become worse than those of the complete HC-SAC, leading to a much lower composite utility. Notably, HC-SAC without hierarchical control obtains a higher secrecy rate than the complete HC-SAC, i.e., 0.516 Mbps versus 0.433 Mbps. This indicates that the non-hierarchical variant tends to learn a more rate-oriented policy, whereas the complete HC-SAC deliberately trades part of the secrecy-rate gain for significantly lower delay and SOP, which is consistent with the overall security–latency trade-off objective. When the constraint-aware learning mechanism is removed, the delay and SOP are improved compared with the standard SAC baseline, but the overall utility is still significantly lower than that of the complete HC-SAC. These observations show that the hierarchical control module and the constraint-aware learning mechanism contribute from different aspects: the former helps handle the coupled UAV, STAR-RIS, and power-control actions, while the latter guides the policy toward lower delay and lower secrecy outage. The complete HC-SAC achieves the highest composite utility of 0.851, suggesting that the two components jointly contribute to the final security–latency trade-off.

### 5.10. Robustness Analysis Under Practical Non-Idealities

To further examine the practical robustness of the proposed method, additional simulations are conducted under imperfect CSI and finite-resolution STAR-RIS phase control. In the imperfect CSI case, the CSI error standard deviation is varied from 0.02 to 0.10. In the finite-resolution case, the STAR-RIS phase resolution is set to 3, 2, and 1 bits. The results averaged over three random seeds are reported in Table 7.

As shown in Table 7, the proposed HC-SAC maintains stable performance under moderate CSI errors and low-resolution phase control. Compared with the ideal CSI and continuous phase case, CSI uncertainty mainly increases the average delay from 10.112 slots to about 10.282 slots, while the secrecy rate, SOP, and successful service ratio remain within a narrow range. The small fluctuations in secrecy rate and SSR under CSI errors are caused by stochastic channel perturbations and should be interpreted as robustness rather than a guaranteed performance gain from imperfect CSI. This limited sensitivity also suggests that the proposed framework, which combines geometry-aware channel features with adaptive resource control, does not rely excessively on highly accurate instantaneous CSI. Moreover, reducing the STAR-RIS phase resolution from 3 bits to 1 bit introduces only a limited performance change at the reference operating point. This indicates that the geometry-aware phase focusing and hierarchical control structure provide certain robustness against practical phase-control quantization. These results indicate stable behavior under the considered non-ideal CSI and finite-resolution STAR-RIS settings.

### 5.11. Reference Performance and Composite Utility Comparison

To provide a concise quantitative summary, Table 8 reports the reference operating-point performance and the AUC-based composite utility of all schemes. The first four metrics are evaluated at the reference operating point, while the normalized composite utility is obtained from the AUC-based evaluation over the corresponding parameter sweeps.

Table 8 summarizes the reference comparison among all schemes. Although PPO achieves the highest average secrecy rate and successful service ratio, HC-SAC provides the lowest delay and the lowest secrecy outage probability. When the four metrics are jointly considered through the normalized composite utility, HC-SAC yields the best overall performance. In particular, compared with PPO, HC-SAC reduces the average delay from 11.7230 to 10.8471 and the SOP from 0.8501 to 0.7160, which leads to a notable composite-utility gain from 0.6630 to 0.9254. These results indicate that the proposed hierarchical and constraint-aware learning strategy provides a more favorable security–latency–service trade-off than both learning-based benchmarks and structural baselines.

## 6. Conclusions

This paper investigated secure and low-latency communications in UAV-mounted STAR-RIS-assisted urban vehicular networks under severe blockage, high vehicle mobility, eavesdropping threats, and delay-sensitive traffic services. A comprehensive system model was developed by jointly considering urban blockage, vehicle mobility, passive eavesdropping threats, queueing dynamics, UAV trajectory evolution, and flight energy constraints. Based on this model, a long-term average secure and low-latency utility maximization problem was formulated and transformed into an MDP with continuous state and action spaces. A hierarchical constrained SAC-based joint control algorithm was then proposed to optimize the UAV trajectory, STAR-RIS transmission–reflection partition ratio, phase-shift matrices, and transmit power allocation.

Simulation results showed that the proposed method achieves lower delay, lower secrecy outage probability, and higher composite utility than DDPG and structural benchmark schemes, while maintaining competitive values for the secrecy rate and successful service ratio. Compared with PPO, the proposed HC-SAC achieves lower delay and lower secrecy outage probability, while PPO retains a higher secrecy rate and successful service ratio. In the representative evaluation, HC-SAC achieves an average delay of 10.8471 slots, an SOP of 0.7160, and the highest normalized composite utility of 0.9254. The ablation study further indicates that both hierarchical control and constraint-aware learning benefit the final security–latency trade-off, since removing either component substantially reduces the composite utility. Additional robustness results show that the proposed method remains stable under moderate CSI errors and finite-resolution STAR-RIS phase control. These results show that HC-SAC does not simply maximize a single communication metric, but achieves a stronger overall security–latency trade-off by reducing delay and secrecy outage probability while maintaining a competitive secrecy rate and successful service ratio.

## Figures and Tables

**Table 1:** Comparison with representative related works.

Work	Main Scenario	UAV Mobility	RIS/ STAR-RIS	V2X	Security	Queue Latency	DRL/ SAC	Hierarchical Constraint Control
[14]	RIS-assisted UAV communication with joint trajectory and passive beamforming design	✓	RIS	–	–	–	–	–
[15]	Performance analysis of RIS-assisted dual-hop UAV communication	✓	RIS	–	–	–	–	–
[16]	Machine-learning-empowered UAV-RIS wireless network	✓	RIS	–	–	–	✓	–
[17]	Robust secure UAV communication with RIS	✓	RIS	–	✓	–	–	–
[10]	DRL-based spectrum allocation and STAR-RIS configuration for V2X	–	STAR-RIS	✓	–	–	✓	–
[12]	UAV-borne STAR-RIS-assisted NOMA communication	✓	STAR-RIS	–	–	–	–	–
[19]	PPO-based RIS-assisted full-duplex 6G-V2X communication	–	RIS	✓	–	–	✓	–
[20]	DRL-based ISAC optimization in RIS-assisted 6G V2X systems	–	RIS	✓	–	–	✓	–
[24]	Improved SAC-based physical-layer eavesdropping defense for V2X	–	–	✓	✓	–	SAC	–
This work	UAV-mounted STAR-RIS-assisted secure and low-latency urban vehicular communication	✓	STAR-RIS	✓	✓	✓	HC-SAC	✓

**Table 2:** Main simulation parameters.

Parameter	Description	Value
*K*	Number of vehicular users	6
*E*	Number of eavesdroppers	1–4
*M*	Number of STAR-RIS elements	64
*N*	Number of time slots	30
$\delta_{t}$	Slot duration	1 s
*H*	UAV flight altitude	80 m
$V_{\max}$	Maximum UAV speed	20 m/s
$P_{\max}$	Maximum transmit power	20–40 dBm
*W*	System bandwidth	10 MHz
$\sigma^{2}$	Noise power	−94 dBm
$\rho_{0}$	Path loss at reference distance	−30 dB
α	Path-loss exponent range	2.1–4.5
κ	Rician factor range	0.08–12
$v_{k}$	Vehicle speed range	6–22 m/s
$\lambda_{k}$	Packet arrival rate	2 packets/slot
$L_{0}$	Packet size	$1.2\times 10^{5}$ bits
$D_{\max}$	Maximum tolerable delay	12 slots
$R_{\mathrm{th}}$	Secrecy outage threshold	0.1 Mbps
$P_{\mathrm{out}}^{\max}$	Maximum tolerable SOP	0.76

**Table 3:** UAV propulsion energy model parameters.

Parameter	Description	Value
$P_{0}$	Blade profile power	79.86 W
$P_{i}$	Induced power in hovering	88.63 W
$U_{\mathrm{tip}}$	Rotor blade tip speed	120 m/s
$v_{0}$	Mean rotor induced velocity	4.03 m/s
$d_{0}$	Fuselage drag ratio	0.6
ρ	Air density	1.225 kg/m^3^
*s*	Rotor solidity	0.05
*A*	Rotor disc area	0.503 m^2^

**Table 4:** Main hyperparameters of the proposed HC-SAC algorithm.

Parameter	Value
High-level actor hidden layers	256-256-128
Low-level actor hidden layers	256-256-128
Critic hidden layers	256-256-128
Activation function	ReLU
Actor learning rate	$1\times 10^{-4}$
Critic learning rate	$1\times 10^{-4}$
Entropy temperature learning rate	$1\times 10^{-4}$
Constraint multiplier learning rate	$1\times 10^{-4}$
Replay buffer size	$1\times 10^{5}$
Mini-batch size	256
Discount factor ζ	0.99
Soft update coefficient ρ	0.005
Initial entropy temperature τ	0.2
Target entropy	−dim(A)
Training episodes	420
Time slots per episode	*N*
Warm-up steps	2200
Evaluation interval	Every 10 episodes
Evaluation episodes	24
Optimizer	Adam

**Table 5:** Benchmark schemes used for performance comparison.

Scheme	Description
Proposed HC-SAC	The proposed hierarchical constrained SAC scheme jointly optimizes the UAV trajectory, STAR-RIS transmission–reflection partition ratio, phase-shift matrices, and transmit power allocation. The hierarchical policy structure decouples large-scale UAV and partition control from fine-grained phase/power control, while the constraint-aware reward shaping mechanism improves the security–latency trade-off.
PPO-based scheme	The proximal policy optimization algorithm is adopted to learn the joint control policy. It uses the same state space, action space, feasibility mapping, reward components, and simulation environment as the proposed HC-SAC. The main difference lies in the on-policy clipped policy update mechanism.
DDPG-based scheme	The deep deterministic policy gradient algorithm is used as another DRL-based baseline. It adopts the same state representation, action mapping rules, reward function, and environmental settings as HC-SAC. Different from HC-SAC, DDPG learns a deterministic policy and does not use entropy-regularized exploration.
Fixed UAV trajectory	The UAV follows a predefined straight-line trajectory, while the STAR-RIS transmission–reflection partition ratio, phase-shift matrices, and transmit power allocation are optimized. This benchmark is used to evaluate the contribution of UAV trajectory optimization.
Fixed STAR-RIS partition	The STAR-RIS transmission–reflection partition ratio is fixed during the whole service period, while the UAV trajectory, phase-shift matrices, and transmit power allocation are optimized. This benchmark is used to evaluate the benefit of adaptive STAR-RIS transmission–reflection partitioning.
Random phase-shift scheme	The STAR-RIS phase shifts are randomly generated, while the UAV trajectory, transmission–reflection partition ratio, and transmit power allocation are optimized. This benchmark is used to evaluate the importance of the STAR-RIS phase-shift optimization.
No STAR-RIS scheme	The UAV provides aerial assistance without STAR-RIS-enabled propagation reconfiguration. Only the UAV trajectory and transmit power allocation are optimized. This benchmark is used to quantify the performance gain brought by the UAV-mounted STAR-RIS.

**Table 6:** Ablation study of the proposed HC-SAC framework.

Variant	Secrecy Rate	Delay	SOP	SSR	Utility
Standard SAC	0.476 ± 0.003	11.753 ± 0.133	0.912 ± 0.004	0.105 ± 0.001	0.146 ± 0.013
HC-SAC w/o hierarchy	0.516 ± 0.008	11.390 ± 0.129	0.880 ± 0.002	0.114 ± 0.002	0.452 ± 0.018
HC-SAC w/o constraints	0.388 ± 0.003	10.831 ± 0.116	0.738 ± 0.002	0.102 ± 0.001	0.552 ± 0.001
Proposed HC-SAC	0.433 ± 0.004	10.440 ± 0.109	0.714 ± 0.002	0.109 ± 0.001	0.851 ± 0.020

**Table 7:** Robustness evaluation of the proposed HC-SAC under imperfect CSI and finite-resolution STAR-RIS phase control.

Setting	Secrecy Rate	Delay	SOP	SSR
Ideal CSI, continuous phase	0.488 ± 0.009	10.112 ± 0.030	0.698 ± 0.002	0.121 ± 0.001
CSI error std. = 0.02	0.492 ± 0.010	10.282 ± 0.049	0.695 ± 0.001	0.123 ± 0.001
CSI error std. = 0.05	0.492 ± 0.010	10.282 ± 0.049	0.696 ± 0.001	0.123 ± 0.001
CSI error std. = 0.10	0.492 ± 0.010	10.282 ± 0.049	0.695 ± 0.001	0.123 ± 0.001
3-bit phase quantization	0.488 ± 0.009	10.113 ± 0.030	0.699 ± 0.002	0.121 ± 0.001
2-bit phase quantization	0.488 ± 0.009	10.112 ± 0.030	0.698 ± 0.002	0.121 ± 0.001
1-bit phase quantization	0.488 ± 0.009	10.111 ± 0.030	0.698 ± 0.002	0.121 ± 0.001

**Table 8:** Reference performance and composite utility comparison of all schemes.

Scheme	Avg. Secrecy Rate (Mbps)	Avg. Delay (Slots)	SOP	SSR	Composite Utility
Proposed HC-SAC	0.3928	10.8471	0.7160	0.1004	0.9254
PPO-based scheme	0.4927	11.7230	0.8501	0.1163	0.6630
DDPG-based scheme	0.4171	11.9358	0.8599	0.1012	0.5060
Fixed UAV trajectory	0.1811	12.4940	0.9058	0.0628	0.2403
Fixed STAR-RIS partition	0.2233	12.2493	0.8932	0.0681	0.3241
Random phase-shift scheme	0.1792	12.4942	0.9036	0.0624	0.2480
No STAR-RIS scheme	0.0903	13.3957	0.9473	0.0497	0.0000

## Data Availability

The code for this paper is available at https://github.com/tangent123/star-ris (accessed on 1 May 2026).

## Abbreviations

The following abbreviations are used in this manuscript:

STAR-RIS	Simultaneously transmitting and reflecting reconfigurable intelligent surface
UAV	Unmanned aerial vehicle
SAC	Soft actor–critic
DRL	Deep reinforcement learning
